# A quantitative interpretation of oxidative protein folding activity in *Escherichia coli*

**DOI:** 10.1186/s12934-022-01982-3

**Published:** 2022-12-22

**Authors:** Lukas A. Rettenbacher, Tobias von der Haar

**Affiliations:** Division of Natural Sciences, School of Biosciences, University of Kent, Canterbury, UK

**Keywords:** *Escherichia coli*, Disulfide bond formation, Oxidative folding, Disulfide proteome, Kinetic modelling, Systems biology, Recombinant protein production

## Abstract

**Background:**

*Escherichia coli* is of central interest to biotechnological research and a widely used organism for producing proteins at both lab and industrial scales. However, many proteins remain difficult to produce efficiently in *E. coli*. This is particularly true for proteins that require post translational modifications such as disulfide bonds.

**Results:**

In this study we develop a novel approach for quantitatively investigating the ability of *E. coli* to produce disulfide bonds in its own proteome. We summarise the existing knowledge of the *E. coli* disulfide proteome and use this information to investigate the demand on this organism’s quantitative oxidative folding apparatus under different growth conditions. Furthermore, we built an ordinary differential equation-based model describing the cells oxidative folding capabilities. We use the model to infer the kinetic parameters required by the cell to achieve the observed oxidative folding requirements. We find that the cellular requirement for disulfide bonded proteins changes significantly between growth conditions. Fast growing cells require most of their oxidative folding capabilities to keep up their proteome while cells growing in chemostats appear limited by their disulfide bond isomerisation capacities.

**Conclusion:**

This study establishes a novel approach for investigating the oxidative folding capacities of an organism. We show the capabilities and limitations of *E. coli* for producing disulfide bonds under different growth conditions and predict under what conditions excess capability is available for recombinant protein production.

**Supplementary Information:**

The online version contains supplementary material available at 10.1186/s12934-022-01982-3.

## Background

Cystine disulfide bonds, covalent connections between the thiol groups of cysteine amino acids, are essential for the correct fold and catalytic activity of many proteins. They are formed by a dedicated cellular machinery, which in native *E. coli* is located in the periplasm [1]. This localisation of the disulfide bond forming machinery, and the high content of reductases and reducing agents such as glutathione in the cytoplasm [2] restrict formation of stable disulfide bonds to the periplasm.

*E. coli* is a commonly used host for recombinant protein expression, but the inability to form stable disulfide bonds in the cytoplasm can restrict its usefulness for expression of recombinant proteins that require such bonds to adopt the correct fold, including many proteins of strong industrial interest like antibody fragments, growth factors, blood clotting factors and enzymes. To enable production of these proteins in a functional form in *E. coli*, either direction to the periplasm is required, or engineering strategies need to be applied that enable disulfide bond formation in its normally strongly reducing cytoplasm. A number of engineering strategies have been proposed, including deletion of the main cytoplasmic thioredoxin reductases [3–5], or expression of recombinant sulfhydryl oxidases and disulfide bond isomerases [6].

Whether disulfide bonds in recombinant proteins are formed by the native *E. coli* machinery upon export to the periplasm, or by engineered pathways in the cytoplasm, host cells must continue the formation of essential disulfide bonds in their native proteins at the same time as meeting the added requirements of recombinant protein expression. To our knowledge, this interaction between the requirements of the native and recombinant proteome has so far not been addressed. Quantifying the normal disulfide bond formation requirements in *E. coli*, relating them to the capacity for disulfide bond formation of the native oxidative folding pathways, and understanding how individual recombinant proteins change this balance of required and provided activity, would enable the further optimisation of engineering strategies for enhancing recombinant protein production in this organism.

The disulfide forming machinery in *E. coli* primarily consists of the ‘Dsb’ family of enzymes. The most abundant of these is the periplasmic DsbA, the cell’s primary thiol disulfide oxidoreductase. DsbA contains a catalytic cysteine bond which can introduce new cystines into unfolded substrates in the periplasm, a process that leads to the reduction of the catalytic cystine in DsbA and the formation of two unpaired cysteines. To regenerate the catalytic activity of DsbA, the cysteine disulfide bond is reformed through an interaction with the periplasmic side of the transmembrane enzyme DsbB, which itself transfers the excess electrons to quinones and eventually to molecular oxygen as the terminal electron acceptor.

In addition to the de novo formation of disulfide bonds, cells require means of correcting proteins in which inappropriately formed disulfide bonds have been formed. DsbA does not possess strong chaperone or isomerising activities, which are instead associated with the dedicated isomerases DsbC and DsbG, which differ in terms of their substrate specificities. Whenever DsbA introduces an incorrect disulfide bond into a substrate, DsbC or DsbG are needed to either reduce the incorrect disulfide or isomerise it to form the correct version. While isomerisation is an electron-neutral reaction, the reduction of a misfolded disulfide bond without its ensuing re-oxidation results in an oxidised, inactive isomerase which can be re-reduced and thereby reactivated by DsbD. In similar fashion to DsbB, DsbD is located in the inner membrane and can facilitate electron transfer; however, in this case it transports electrons into the periplasm. The cytoplasmic side of DsbD can transfer a disulfide bond on to thioredoxins which in turn allows DsbD to accept an excess disulfide bond from the isomerases.

The different enzymes of the disulfide forming machinery all have different concentrations and enzyme kinetics. This machinery introduces disulfide bonds into a wide range of host substrates, including recombinant proteins where these contain disulfide bonds. Moreover, differing growth conditions can impact on the oxidative folding machinery as well. The dynamic interactions in this complex system have so far not been fully addressed experimentally. Here, we develop computational models of the oxidative folding process in *E. coli* and use proteomic data to estimate the relationship between provided and required activity under different growth conditions, and to describe and predict the impact of cell engineering and recombinant protein production on the native disulfide machinery.

## Results

### A quantitative estimation of the oxidative folding machinery in *E. coli*

An initial aim in this study was to estimate the required rates of de novo disulfide bond formation and disulfide bond isomerisation in *E. coli* on the one hand, and the abundance of components of the oxidative folding machinery and the enzymatic activity they provide on the other. We then set out to bring these two elements together by using a quantitative modelling approach to describe the oxidative folding system dynamically.

We initially collected a total of 73 quantitative *E. coli* proteomes from seven different publications [7–13]. Six of these publications provide absolute protein quantification values in the form of protein copy numbers per cell. A seventh study by Peebo et al*.* provides protein concentrations, which we converted to protein copy numbers per cell by estimating the cell size from data provided in the study as explained in the Materials and Methods section. The collected proteomes cover a variety of growth conditions, differing in media composition, carbon sources, growth rates, *E.coli* strains and stresses imposed on the cells. To select the most suitable datasets for modelling, we analysed the proteomes in terms of their quantitative protein coverage as well as completeness of the additional information provided with the proteomic data. In a first step we excluded proteomes without reported growth rates since this information was essential for estimating protein synthesis rates (see below). In order to estimate proteome coverage in these studies, reported values for total cellular protein count were compared to the corresponding theoretical total cellular protein count based on published calculations [14], which exploit the fact that cellular protein count correlates with both cell size and cellular growth rates. Growth rates were used to estimate the corresponding cell sizes using Eq. (1). The resulting estimates of fractional proteome coverage are displayed in Additional file 2: Fig S1. Proteomes with a quantitative coverage below 50% were not considered for the quantitative modelling part.

Good overall proteome coverage is important for good representation of oxidative folding substrates in the datasets. In addition, we intended to use the datasets also as a source for evaluating the abundance of oxidative folding enzymes, and we therefore specifically investigated how well the Dsb enzymes were represented in them.

**Fig. 1:**
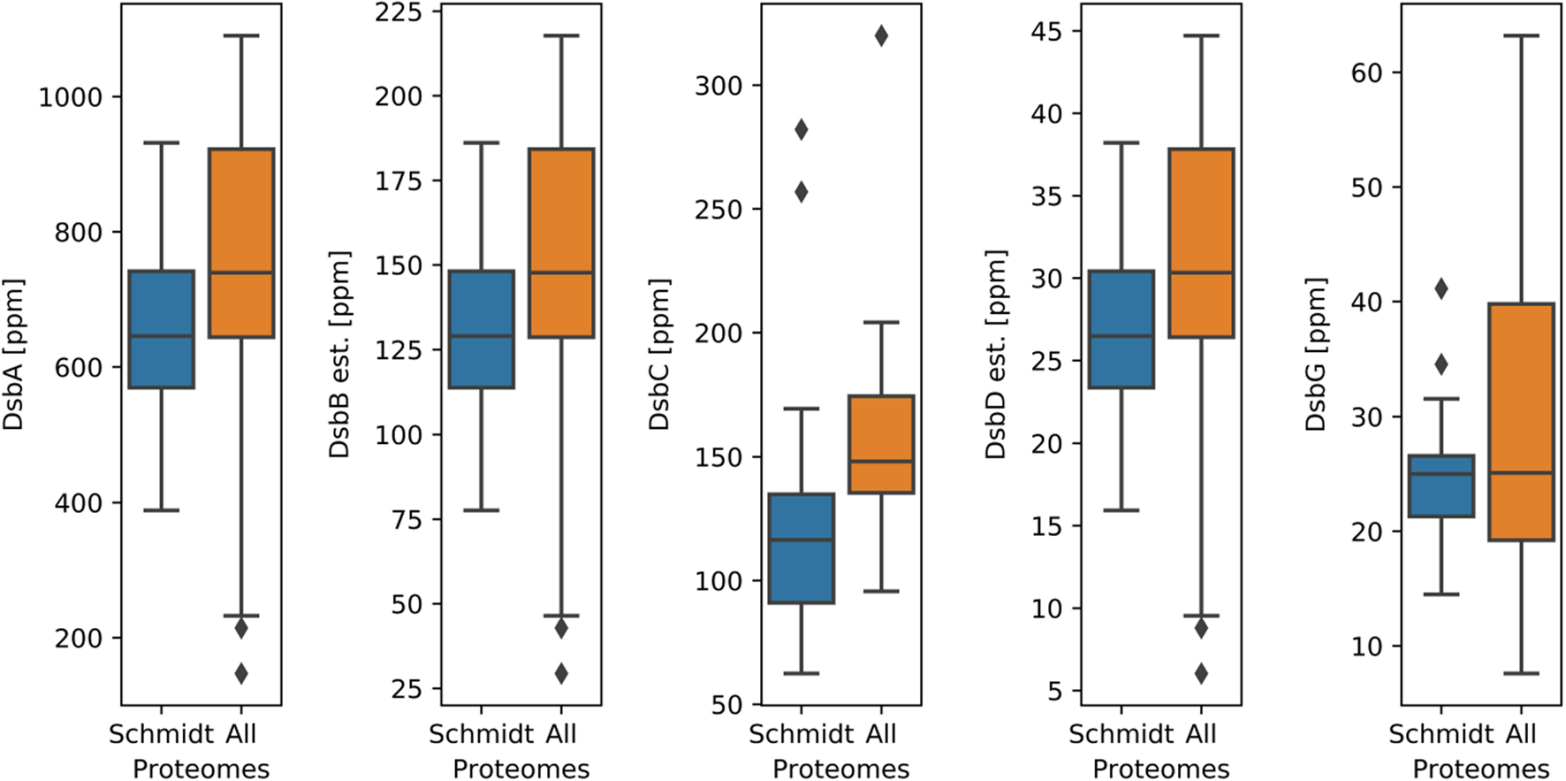
Boxplots displaying the concentration ranges for the oxidative folding enzymes. For the membrane proteins DsbB and DsbD the estimated concentrations are displayed (DsbBe and DsbDe). The concentrations are given in parts per million (ppm), i.e. enzyme count per million host cell proteins. On the left (blue) the concentration ranges based on the quantitative proteomes by Schmidt et al. [13] are displayed. On the right (orange) the whole range of concentrations from all collected quantitative proteomes are displayed

The primary oxidase DsbA is an abundant, soluble protein detected in all datasets with a mean abundance of 696 ppm (proteins per million proteins) or around 4000 proteins per cell (Fig. 1). The two isomerases DsbC and DsbG are also soluble proteins. The more abundant DsbC was again represented in all datasets with a mean concentration of 144 ppm, or 800–900 proteins per cell. The less abundant DsbG was not represented in two of the datasets, but in those datasets where it was represented the concentration was reported with a mean abundance of 27 ppm or around 160 proteins per cell. In contrast to the good representation of the soluble enzymes, the membrane-bound DsbB and DsbD had no associated abundance data in the majority of studies, only being covered in 25 and 23 of the 73 datasets, respectively. This was expected, since membrane-associated proteins are frequently under-reported in proteomics datasets if not specifically accounted for during sample preparation [15]. The large whiskers of the boxplots shown in Fig. 1 demonstrate how heterogeneous the observed levels of these enzymes can be. This relatively large variance is in part derived from variations in measurement of the different proteomes, but also from different protein expression levels under different growth conditions.

Because of the poor representation of the membrane-associated oxidative folding enzymes, we estimated their abundance from synthesis rate data reported by Li et al. [16]. This study used a ribosome footprinting approach to characterise protein synthesis activity in the *E. coli* translatome. By determining apparent synthesis rates for all Dsb proteins, we were able to establish a relationship between synthesis rates and steady state levels for DsbA, C and G, which we then used to predict steady state levels for DsbB and D from their synthesis rates. These analyses yielded mean concentrations of 139 ppm and 29 ppm for DsbB and DsbD, respectively (corresponding to around 480 and 100 proteins per cell).

Based on the overall quantitative coverage analysis, the availability of additional information regarding growth rates, cell size and stress conditions as well as the coverage of the key ‘Dsb’ enzymes, the proteomes reported by Schmidt et al. [13] were selected for the rest of this study (unless noted otherwise). Additionally, only disulfide bonds and the enzyme functions associated with their formation were considered in this study. Other cysteine modifications such as sulfenic acids and their reduction via DsbC or DsbG were not included in this analysis [17].

### The *E. coli* disulfide proteome

Following initial quality controls and selection of suitable datasets, we used the proteomics data do estimate the volume of disulfide bonds processed by the Dsb enzymes in native *E. coli* cells. Proteins are substrates if they are located in the periplasm and contain disulfide bonds in their folded state, and we used ancillary data sources to identify the subset of cellular proteins to which these criteria apply.

**Fig. 2:**
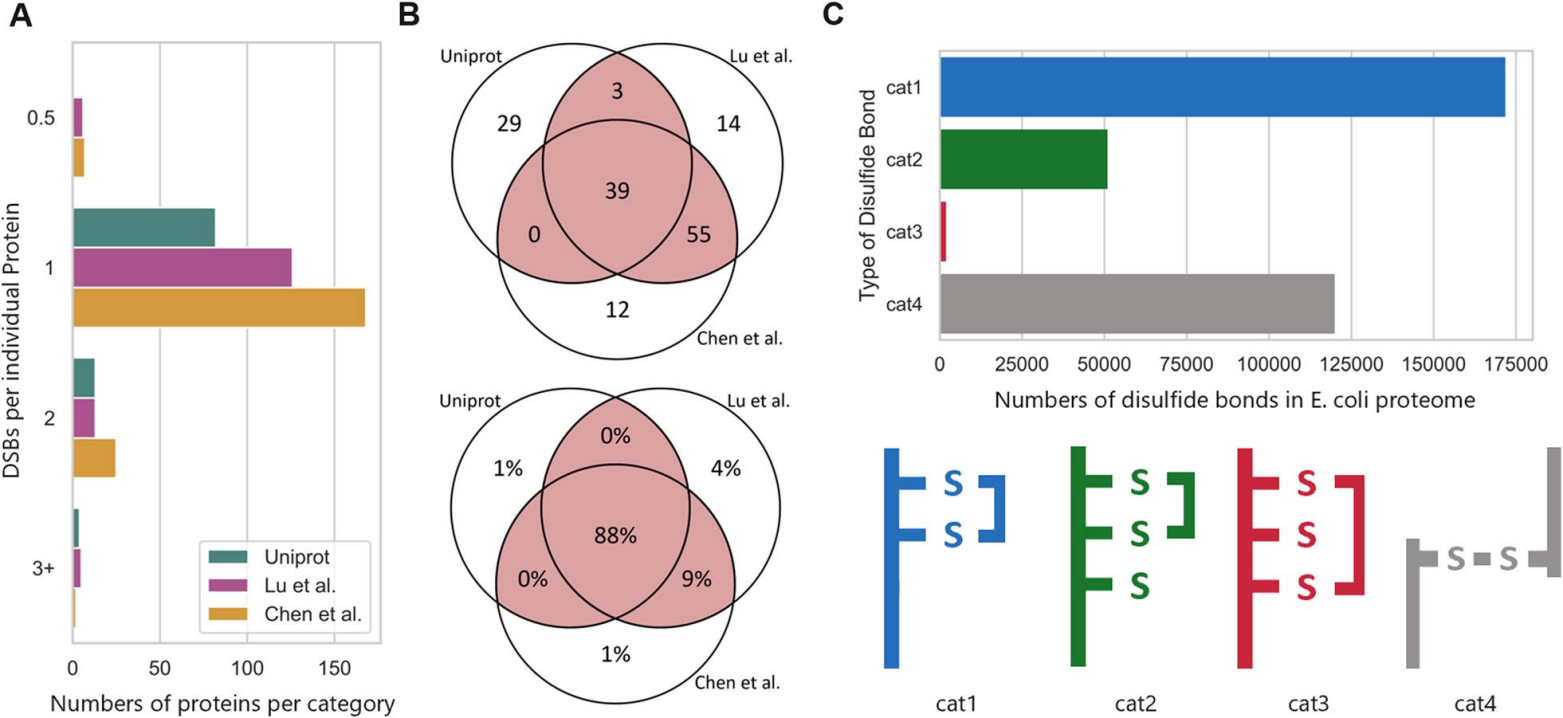
Summary of the disulfide bond composition of the E. coli proteome. **A** Number of disulfides per protein per literature source. “0.5” refers to intermolecular disulfide bonds. Not shown is one protein with 2.5 disulfide bonds identified in Chen et al. **B** Numbers of disulfide bond containing periplasmic proteins (top) and estimated total numbers of periplasmic disulfide bonds (calculated by multiplying the numbers of disulfide bonds in each protein with protein abundance during growth on glucose, bottom) in three data sources. Disulfide bonds identified by 2 + sources are highlighted in red. Areas in the Venn diagram are not proportional to numbers. **C** Number of disulfide bonds per “folding difficulty” category

Three different sources of known disulfides in the *E. coli* proteome were considered to identify disulfide-bond containing proteins: The Uniprot database [18] which contains curated annotations from the research literature and two proteomics studies [19, 20] which used labelling techniques to identify native *E. coli* disulfide bonds by mass spectrometry. The three data sets collectively identified 360 distinct disulfide bonds that can form in *E. coli* proteins (Fig. 2A); however, only 45 of these were identified in all three (39 of which are periplasmic, Fig. 2B). These initial observations suggest considerable residual uncertainty when it comes to the *types* of disulfide bond that can form in *E. coli* proteins. However, agreement between the studies is better for highly expressed proteins, and in consequence the uncertainty regarding the total *number* of disulfide bonds that are formed in bacterial cells is much lower (Fig. 2B).

To identify proteins located in the periplasm, we used data generated by Loos et al. [21] who trained a machine learning algorithm to annotate protein locations based on a protein’s primary amino acid sequence, which suggests locations for 98% of all known *E. coli* proteins with high confidence. We considered all proteins annotated in this dataset as ‘secreted’ and ‘secreted outer membrane’ as potential substrates for the Dsb machinery. An overview of numbers of periplasmic disulfide bonds identified in this way is presented in Fig. 2.

A closer look at the reported disulfide bonded proteins confirms the common assumption that *E. coli* has a relatively simple disulfide proteome (Fig. 2A). The total set of 360 reported disulfide bonds are located on 285 different proteins, with only 18 proteins having more than one potential disulfide bond. 174 of the 360 identified disulfide bonds can be allocated to either the periplasm or the outer cell membrane. Of those, 140 are formed by consecutive cysteines, 12 by non-consecutive cysteines and 22 are intramolecular. Out of the 174 disulfides in *bona fide* periplasmic proteins, 97 are described by 2 + sources, and we used this “higher confidence” subset for the kinetic modelling studies described in the following (Fig. 2B). This set of 97 disulfide bonds is located on 82 individual proteins, with only 6 proteins having more than 2 potential disulfide bonds. The list of all 360 identified disulfide bonds and their protein IDs is provided in the Additional file 1: (sheet 2—‘DSB data’).

The disulfide bond datasets provide a static picture of disulfide bonds in the *E. coli* proteome. However, it does not yet incorporate information on the folding pathways by which individual disulfide bonds are formed, and in particular whether correct bonds are formed immediately through the action of DsbA or following initial incorrect formation and subsequent isomerisation by DsbC or DsbG. To our knowledge there is no quantitative, proteome-wide information available to address this question. We therefore introduced a number of semi-quantitative assumptions that we then used to formulate boundary conditions for required levels of isomerase activity in the cell. We categorised disulfide bonds into four categories which we assume are of increasing risk of misfolding, based on relative cysteine locations on each protein’s amino acid chain. The first category contains proteins that only have two cysteines, where no mispairing is possible. The second category contains proteins where disulfide bonds are formed between consecutive cysteines, but where additional cysteines exist that could mis-pair with either of the cysteines involved in the native bond. Thirdly we consider disulfide bonds between non-consecutive cysteines, where we assume a more substantial risk of incorrect disulfide bond formation with the intervening cysteine. A fourth category contains intermolecular disulfide bonds and was not further considered in this analysis.

A quantitative evaluation of proteins and associated disulfide bonds in each “folding difficulty” category is shown in Fig. 2C. This graph displays total numbers of disulfide bonds in each category, calculated as the number of proteins multiplied with each protein’s abundance. Overall the risk of disulfide-bond related misfolding in *E. coli* appears relatively low, since two thirds of disulfide bonded proteins contain exactly the two cysteines required for the disulfide bond to form, without any scope for misfolding. This finding is consistent in principle with the observation reported above that the enzymes involved in disulfide bond isomerisation (DsbCDG) are expressed at much lower levels compared to DsbAB.

### Modelling oxidative folding and isomerisation

**Fig. 3:**
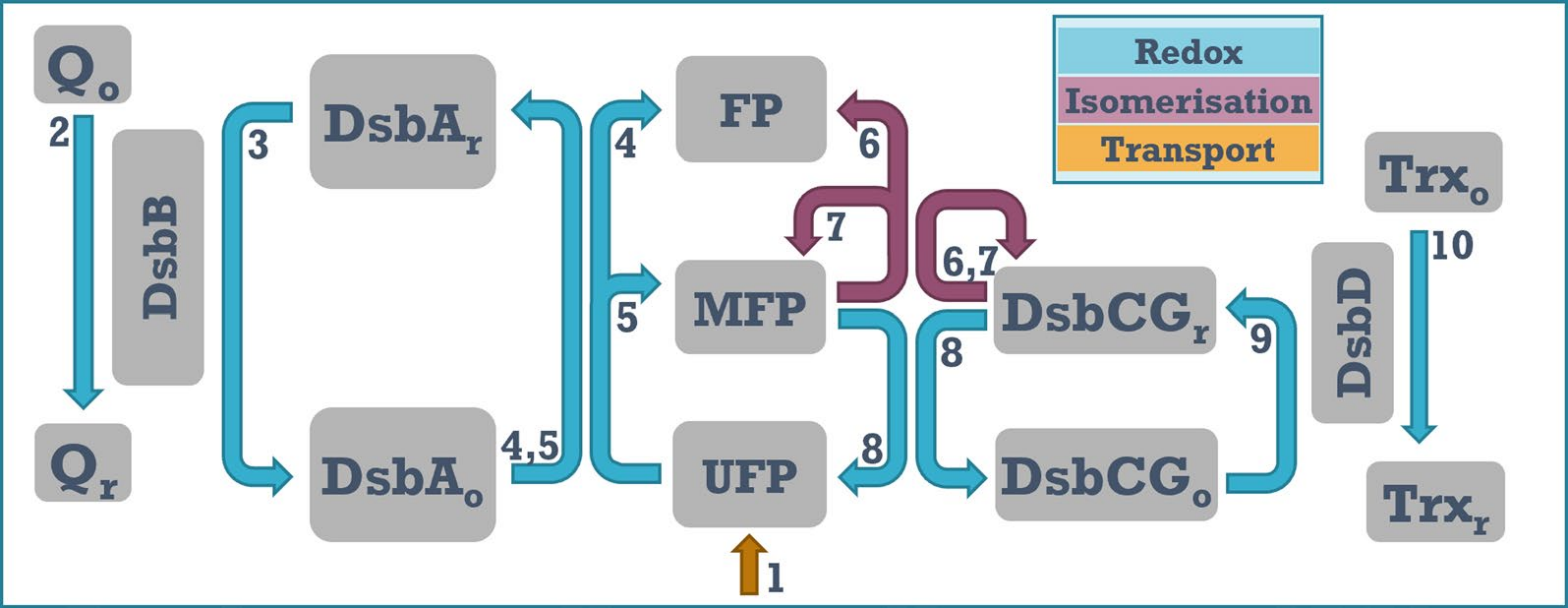
The oxidative folding model. (1) Synthesis of proteins with a reduced disulfide bond. In the model there are three synthesis rates, one for each difficulty category, and synthesis rates are estimated from the known steady-state abundance of proteins in each category and the growth rate. (2) Oxidation of DsbB via quinone. (3) Oxidation of DsbAr by DsbB. (4) Correct substrate oxidation by DsbAo. (5) Incorrect substrate oxidation by DsbAo. (6) Correct substrate isomerisation by DsbCGr. (7) Incorrect substrate isomerisation by DsbCGr. (8) Reduction of wrongly oxidised substrates by DsbCGr. (9) Reduction of DsbCGo by DsbD. (10) Reduction of DsbD by Thioredoxin

To investigate the dynamics of periplasmic oxidative folding processes in *E. coli,* we used an ODE-based computational model with a reaction scheme as depicted in Fig. 3. The model assumes a steady influx of folding substrates into the periplasm, where the substrates are grouped into the different folding categories shown in Fig. 2. The rate of substrate influx into the periplasm is estimated from the quantitative disulfide proteome and the cells’ growth rate, which gives the minimum rate of protein synthesis required to maintain a stable proteome in the steady state. In reality additional protein synthesis is required to counteract protein turnover, but under rapid growth conditions this proportion is small compared to that required due to growth [22].

In the model, proteins enter the periplasm in a reduced and unfolded state (*UF*) but can be oxidised by interaction with DsbA_O_. The outcome of this oxidation can be either the adoption of a misfolded (*MFP*) or a correctly folded (*FP*) state. We assume that the reaction kinetics leading from *UF* to *FP* and *MFP* are identical, but that the probabilities of immediately adopting the *FP* state differ for the three substrate classes, being 100% for ‘cat1’, 50% for ‘cat2’ and 0% for ‘cat3’. Although actual proteins are more likely to form a continuum of folding probabilities, we assume that these discrete protein categories in the model capture the different types of behaviour observed in biological proteomes both in terms of the types of reactions that occur and (in a first approximation) in terms of their quantitative requirements of de novo folding and isomerase activities provided by the *E. coli* oxidative folding pathways.

Following oxidation of *UF* proteins, DsbA is reduced and can be regenerated by DsbB, which in turn is regenerated in a redox reaction with quinone. If *UF* oxidation leads to formation of *MFPs*, these can further interact with the isomerase DsbCG where one of three things can happen: (1) the disulfide bond is successfully isomerised to form *FP*, (2) the isomerisation is unsuccessful and the protein remains in the *MFP* state or (3) the disulfide bond on the substrate is reduced by the isomerase, returning the protein to the *UF* state. In the latter case, the isomerase itself becomes oxidised and has to be regenerated via DsbD, which in turn transfers the excess electrons onto thioredoxin. In the model representation in Fig. 3, quinones and thioredoxin are depicted in the periplasm for simplicity, even though in reality these reactions take place on the cytoplasmic side of the transmembrane proteins and the inner cell membrane respectively. However, since the model simplifies the reoxidation of DsbB and the reduction of DsbD into pseudo-first order reactions, the actual location of these terminal components is irrelevant in this context.

Each model reaction is represented by an ordinary differential equation and has an associated kinetic parameter. These kinetic parameters dictate the speed of each reaction, and in cases where there is more than one possible reaction outcome, also the ratio between the possible outcomes.

### Kinetics

We initially parameterised the model using enzyme concentrations derived from the quantitative proteomics data, and substrate concentrations derived from proteomics data covering disulfide-bonded proteins in the different “folding difficulty” categories outlined above. We assume that clients of the Dsb proteins are predominantly newly translated proteins which are not yet correctly folded. The rates with which such Dsb clients are generated can be estimated from their cellular abundance in the steady state and from the growth rate, since the rate of growth dilution dominates rates of protein turnover in fast growing microbial cells [23].

Based on these known substrate production rates, we then characterised minimal enzyme rate constants that were compatible with the essential requirement of doubling the *E. coli* proteome once per generation. This strategy allows estimating minimal required enzyme activities for the core reactions in the model but may underestimate the actually required activity if futile cycles occur frequently. For example, if a disulfide bond formed by DsbA is resolved again by DsbCG, or if a protein in a mis-folded state is simply transferred to another mis-folded state rather than the correctly folded one, enzyme activity is engaged without a net change in substrate or product concentrations. Because we have no information allowing us to estimate the frequency of such cycles, we assumed here that such cycles are rare compared to productive folding events. In our model parameters, we assumed that futile cycles make up one third of all isomerase-catalysed reactions which we considered to be a conservative if not over-estimation of the futile reactions taking place in the cell.

We applied this strategy to all datasets generated by Schmidt et al*. *[13], thus generating specific minimally required enzyme rate constants for each of the growth conditions investigated in this study. Due to the specific reaction structure employed in the model, the results are returned in the form of apparent association rate constants for the formation of enzymes–substrate complexes. It is worth noting the relationship between these reported apparent rate constants and the actual enzyme rate constants: because we characterise *minimal* rate constants required for the system to cope with the observed substrate influx, these are expected to be slower than actual biochemical enzyme rate constants if an enzyme is not engaged at its maximum capacity. On the other hand, the modelled apparent rate constants cannot be faster than the actual rate constant as this would be biochemically impossible and would indicate that either enzyme or substrate concentrations have been reported incorrectly, or that the model structure has been chosen inappropriately.

To facilitate interpretation, we multiplied the enzyme concentrations for each condition with the modelled apparent association rate constants, thereby creating a pseudo first-order rate constant expressing how rapidly substrates are likely to be processed in each of the different growth conditions (Table 1). Lower first-order rate constants indicate that enzymes engage less readily with their substrate, because in the respective condition the ratio of provided to required activity is lower. In terms of the question we initially asked, high rate constants thus imply a degree of oversupply in the system, which could be exploited for example for more efficient recombinant protein processing.

**Table 1:** Reaction rates for oxidative folding in *E. coli*

	Reaction(s)						
	R2	R3	R4 (Cat1)	R4,5 (Cat2)	R5 (Cat3)	R6,7,8 (Cat2)	R6,7,8 (Cat3)
Chemostat							
Chemostat µ = 0.5	0.090	5.388	0.946	0.433	1.297	0.227	0.527
Chemostat µ = 0.35	0.091	4.526	1.093	0.703	2.106	0.673	1.957
Chemostat µ = 0.20	0.040	3.943	0.791	0.535	1.603	0.295	0.886
Chemostat µ = 0.12	0.016	3.181	1.367	0.924	2.770	0.185	0.557
High Growth							
LB	0.228	6.727	1.894	0.337	1.131	0.246	0.584
Glycerol + AA	0.199	6.708	2.519	0.471	1.151	0.364	0.371
Stress							
pH6 glucose	0.152	7.571	1.599	0.733	2.084	0.227	0.486
42 °C glucose	0.048	6.801	1.023	0.370	1.108	0.112	0.240
Osmotic-stress glucose	0.111	6.637	1.107	0.482	1.518	0.192	0.412
Non-stress, sub-optimal							
Acetate	0.046	2.758	0.613	0.394	1.181	0.100	0.299
Fructose	0.079	3.230	1.061	0.425	1.149	0.187	0.301
Fumarate	0.065	3.888	0.988	0.556	1.664	0.158	0.406
Galactose	0.044	3.549	0.857	0.562	1.737	0.165	0.505
Glucosamine	0.083	5.008	1.005	0.537	1.608	0.210	0.515
Glucose	0.080	4.309	0.796	0.404	1.092	0.168	0.292
Glycerol	0.048	5.183	0.892	0.486	1.426	0.122	0.321
Mannose	0.072	3.599	0.722	0.320	0.960	0.119	0.321
Pyruvate	0.042	3.351	0.896	0.576	1.726	0.104	0.312
Succinate	0.065	3.925	0.873	0.481	1.440	0.176	0.453
Xylose	0.077	4.400	0.772	0.413	1.059	0.145	0.344
Median	**0.07**	**4.35**	**0.97**	**0.48**	**1.43**	**0.18**	**0.41**
Range (fold)	14.3	2.7	4.1	2.9	2.9	6.7	8.2
Inter quartile range (fold)	1.9	1.6	1.3	1.4	1.5	1.6	1.6

We observed significant variation in oxidative folding capacity between the different growth conditions (Table 1). The de novo folding reactions (R4,5 in Fig. 3) vary over a two- to four-fold range between conditions, and the isomerisation reactions vary over a six- to eight-fold range. As the demand for oxidation and isomerization changes, so does the demand on the enzymes that catalyse these reactions. What we observe here is that the range of demand for the oxidative system is lower compared to the range of demand on the isomerization system. Each of the investigated proteome datasets reflects specific combinations of growth rates, oxidative folding enzymes and their substrates, and the observed folding capacity most likely changes as the result of relative changes in these parameters.

**Fig. 4:**
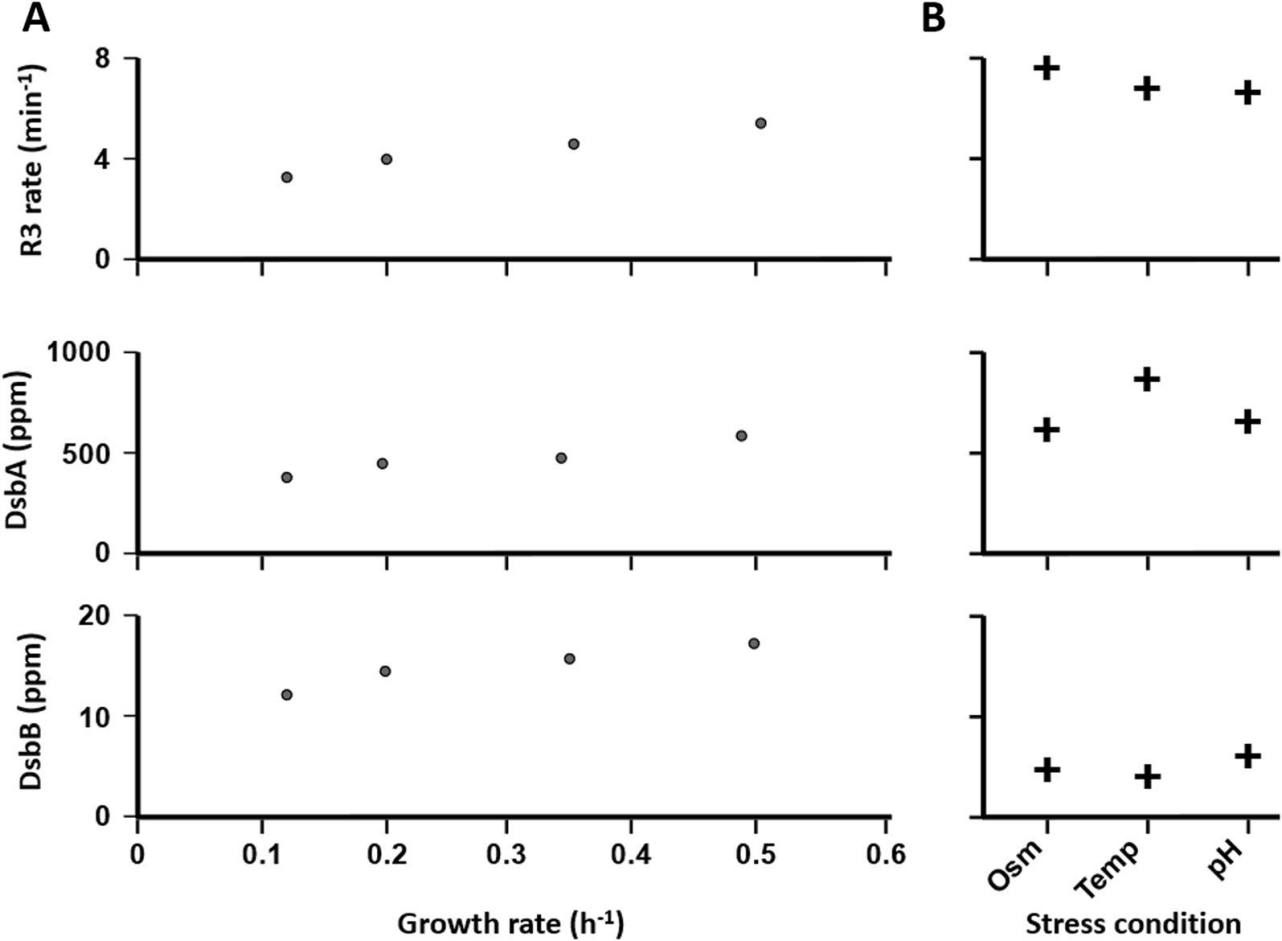
The rate of DsbB- catalysed reoxidation of DsbA and DsbA/B enzyme abundance under different growth conditions. **A**, during chemostat growth the abundance of DsbA, DsbB and the capacity for DsbA-reoxidation by DsbB (represented here by the modelled R3 rate) all correlate positively with growth rates. **B**, under stress conditions, DsbA abundance and DsbB capacity are high despite low DsbB enzyme concentration

We observed a clear dependence of the capacity for DsbA reoxidation by DsbB with growth conditions. The highest capacities for this reaction, with substrate processing rates of 6 min^−1^ and higher, were observed during stress conditions which in this dataset included low pH, high temperature and osmotic stress; as well as growth in LB and amino-acid supplemented glycerol, two non-stress conditions with high growth rates. Moreover, the chemostat series of experiments, in which growth rates are directly controlled by the dilution rate with otherwise identical parameters, revealed a strong correlation between the capacity to regenerate DsbA and growth rates (Fig. 4A. Pearson’s Product-Moment Correlation Coefficient for the correlation between growth rate and R3 rate parameters for this reaction is 0.98). None of the other reaction rates show similar patterns, and in particular the de novo folding reactions (R4,5) show no clear correlation with the same conditions. Indeed, the majority of the apparent R4/5 rates appears remarkably constant with the lowest inter-quartile range of all reactions. One interpretation of these findings is that *E. coli* cells adjust the expression and subsequent availability of DsbA, including the cells’ ability to reactivate this enzyme, in line with demand arising from increasing growth speed, thus enabling the timely processing of inflowing substrates.

Interestingly, the maintenance of DsbA capacity under high growth and stress conditions appears to be the result of distinct set-ups of the oxidative folding machinery (Fig. 4). During chemostat growth, the abundance of both DsbA and DsbB increases, resulting in an overall increase in the capacity to regenerate DsbA (Fig. 4A). This scenario is consistent with an increasing need for de novo folding in response to the increased dilution rates during fast growth, when the influx of DsbA substrates increases proportionally with growth and dilution rates. Under such conditions, both DsbA and the capacity to regenerate this enzyme must be adjusted concomitantly. In contrast, the high levels of DsbA under stress conditions appear to be efficiently reoxidised (they have high R3 rates) despite low DsbB concentrations. As the overall capacity of DsbA to catalyse de novo folding is still high under stress conditions compared to normal growth (see apparent R4 rates in Table 1), the most parsimonious explanation is that during stress high DsbA levels are maintained despite relatively low de novo folding demand.

### Oxidative folding demand and recombinant protein production capacity

**Fig. 5:**
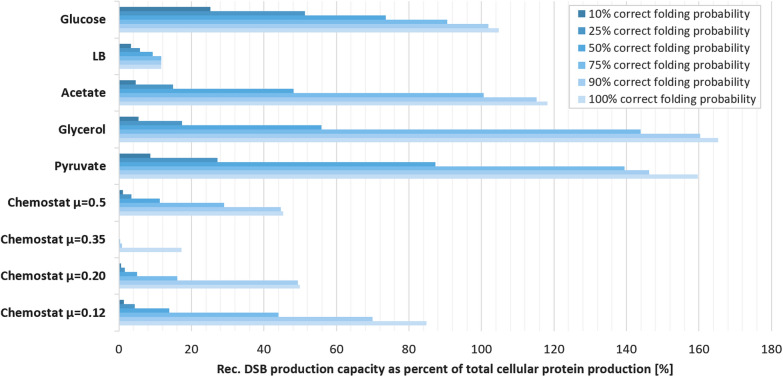
Predicted recombinant protein production capacity for different growth conditions. Estimated by introducing a theoretical recombinant disulfide bonded protein into the model and simulating its impact on the host proteome in terms of oxidative folding. The calculated synthesis rates are shown as a percent of the overall protein production rate of the cell at each given growth condition. Results shown for different disulfide bond folding complexities of the recombinant protein

In addition to analysing the native capacity for oxidative folding in *E. coli*, the model allows estimating the capacity of this organism for dealing with additional substrates such as recombinant proteins. We investigated this by increasing the production rate for oxidative folding substrates of varying “folding difficulty”, i.e., where the probability of immediately adopting the correct fold decreases and the probability of adopting an incorrect fold which needs to be further corrected by an isomerase increase. We assume that there is no regulatory adaptation to the new substrate. In order to monitor the capacity to process recombinant substrates under particular growth conditions, we increase the rate with which additional recombinant proteins are produced until the cells’ native substrates begin to accumulate (we use an accumulation-threshold of the host cell disulfides of 0.5%- as a cut-off for determining the point at which the recombinant protein production starts to impact the upkeep of the host proteome). The amount of recombinant protein that can be introduced before this threshold is reached is displayed in Fig. 5.

The results suggest that the capacity to produce recombinant protein varies strongly with growth conditions, as well as with the requirement for isomerisation. Growth in glycerol, glucose and pyruvate are predicted to allow the highest yields in principle, with an estimated capacity of processing up to 150% of recombinant protein over and above the normal cellular protein complement. When isomerisation steps are required, the yield is predicted to drop strongly, with very-difficult-to-fold proteins that rely highly on isomerase activity showing only a fraction of the predicted yield of an otherwise equivalent easy-to-fold protein. Interestingly, the capacity to process isomerase-requiring substrates differs more strongly between conditions than non-isomerase-requiring ones, and the relationship is not proportional: for example, during growth in glucose the capacity to process an easy-to-fold substrate is predicted to be less than during growth in glycerol, but the capacity to process a difficult-to-fold substrate is 4–5 times higher during growth in glucose. We assume that these differences reflect different proportions of DsbAB vs DsbCDG concentrations, and indeed examinations of the proteome datasets reveals that whereas concentrations of most Dsb enzymes are comparable under both growth conditions, the concentration of DsbC is increased during growth in glucose.

## Discussion

Our model-based investigation of the disulfide-bonded proteome in *E. coli* suggests that cells use different strategies for providing the required oxidative folding capacity under different growth conditions (Table 1 and Fig. 4). Under non-stress conditions, concentrations of DsbA and DsbB appear to be adjusted strictly in parallel, both increasing with faster growth and the resulting requirement for faster processing of de novo folding substrates (Fig. 4). This results in the provided DsbA activity remaining well matched with requirements, as indicated by the relatively low inter quartile range for modelled de novo folding rate constants during non-stress growth (Table 1). During stress conditions, the cellular strategy appears to differ substantially from the non-stress one in that here atypically high DsbA concentrations coincide with atypically low DsbB ones. The elevated DsbA:DsbB ratio should result in an increased cellular concentration of reduced DsbA. This could benefit the cell by facilitating a less ‘generous’ substrate oxidation strategy, only providing disulfide bonds to certain, high affinity substrates. The elevated levels of reduced DsbA could also help alleviate the mis-oxidation stress of the cell by acting as a disulfide bond acceptor for mis-oxidized substrates. An alternative explanation for this elevated DsbA:DsbB ratio could be that cells are preparing for a “growth ready” strategy: this could provide sufficient DsbA to rapidly cater for folding demand when growth rates increase again following stress recovery, then only requiring adjustment of DsbB levels which are much lower than for DsbA and can therefore be increased relatively more quickly.

Although the modelling outputs presented in Table 1 are meant to indicate comparative activity between different growth conditions only, it is useful to ask how they relate to the biochemical rate constant reported in the literature. Darby and Creighton reported biochemical assays in which they used DsbA to fold the three disulfide bond-containing Bovine Pancreatic Tryspin Inhibitor (BPTI) [24], where they observed initial association rate constants above 10^5^ M^−1^ s^−1^ whereas the release into the MFP or FP product occurred at estimated rates of 2.7 s^−1^. DsbA concentrations estimated from the proteome datasets are between 5 and 30 µM under all growth conditions, so that formation of the catalytic complex would typically be rate limiting. Interestingly, the minimally required de novo folding rates we report in Table 1 (R4 and R5) are typically around 1 s^−1^, whereas the equivalent actual rates revealed by the biochemical experiments would be between 0.5 s^−1^ at 5 µM and 3 s^−1^ at 30 µM DsbA concentration. These analyses assume that DsbA is maintained in a mostly or wholly oxidised state. If, under steady state conditions, a substantial proportion of DsbA was in a reduced state awaiting reoxidation, the ability to catalyse *do novo* folding would be reduced proportionally. Overall, the comparison to available biochemical data suggests that the minimally required and actually provided DsbA capacity is within one order of magnitude and support the notion that DsbB concentrations need to be adjusted in concert with DsbA levels in order to maintain sufficient continuous DsbA activity.

In previous work, Karyoleimos et al*.* investigated how different recombinant protein production rates affected steady-state expression levels of secreted single chain antibody (scFv) and human growth hormone (hGH), two recombinant proteins with differing disulfide bond patterns [25]. This study reported that gene expression pathways became quickly overwhelmed as expression levels increased, and that efficient processing of the recombinant proteins required adjusting expression levels to the lower range of the rhamnose-inducible system used in that study. While this study focused on the limiting capacity of the Sec translocon as the main bottleneck for the production of periplasmic recombinant proteins, some of the presented data indicate that there are concurrent issues with protein folding, including the apparent inability of the Dsb machinery to produce fully disulfide bonded hGH when expression levels were adjusted to allow for most efficient secretion (cf. Figure 5 B in Karyoleimos et al*.* 2019). One of the testable predictions resulting from our analyses is that adjusting growth media could be a viable strategy for adjusting the available oxidative folding capacity to the needs of individual recombinant proteins, by allowing to adjust both the overall folding capacity and the ratio of *do novo* folding to isomerase activity (Fig. 5). Our results suggest that the ‘ideal’ growth conditions for recombinant disulfide bond formation depends strongly on the type of disulfide bond(s) required by the target protein. For ‘simple’ disulfides, which rely solely on the oxidative folding machinery, growth on glycerol with a moderate growth rate (~ 0.5 µ) appears favourable based on our modelling results. For recombinant proteins with complex disulfide pattern, glucose-based growth with a moderate growth rate (~ 0.6 µ) results in the best yield prediction. While fast growing cells on LB media (1.9 µ) exhibit a low excess capacity for oxidizing recombinant proteins, the highly elevated biomass formation can compensate this disadvantage. In cases where volumetric yields are more important than effective C-source usage, this growth strategy is also predicted to yield good results for both complex and simple disulfide patterns. Chemostat growth seems to be unsuited for the efficient production of disulfide bonded proteins compared to the other observed growth conditions. However, given the relatively close match between required and provided Dsb activity, the success of such media-based strategies will likely remain limited and substantial increases in oxidative folding capacity would require the introduction of engineered systems such as *CyDisCo *[26], which provides oxidative folding capacity in the cytoplasm thereby circumventing both Sec and Dsb bottlenecks.

## Conclusion

In summary, our study shows that the combination of genome-wide datasets and modelling approaches can be used to explore feasible rate constants even when information on the actual biochemical rates in a system is limited. This approach is particularly useful for estimating the capacity of cell-wide pathways to cope with both endogenous demand and any additional demands arising from bioprocessing, bioengineering or synthetic biology needs, and the resulting information can be used to inform strain and process engineering strategies to optimise relevant cellular pathways.

## Materials and methods

### Datasets

Data manipulations including cleaning and merging of different data sources were performed using the Python numpy [27] and pandas [28] libraries.

The quantitative proteomes used in this study were extracted from Additional files available with the relevant literature [7–13]. Information regarding strain types, growth conditions and protein quantification methods were also extracted from these publications. The quantitative data sets from these publications were merged into a single data table based on individual proteins’ Uniprot IDs.

To assess the number of *potential* disulfide bonds per protein in the *E. coli* proteome, protein sequences were downloaded from Uniprot [18] and the maximum number of cysteine pairs was calculated as the number of cysteines divided by two, rounded down to the nearest integer.

*Actual* numbers of disulfide bonds per protein were collected by merging three experimental data sources. One was derived from annotated disulfide bonds in the uniport database. The other two were based on disulfide-labelled proteomes. All three data sources identify the specific cysteins in the protein sequence which form disulfide bonds and the data sets were merged based on these cysteins and the corresponding protein IDs. In cases were a single cystein can form disulfide bonds with different partners, only a single disulfide was considered for the evaluation of the cellular oxidative folding requirement. In cases were a cystein forms an oxidative bond with itself (on another copy of the same protein), the disulfide bond was counted as 0.5 for the quantitative evaluation. Disulfide bonds identified between different proteins were not considered in this analysis.

The classification into “folding difficulties” (see the Results section for details) was then performed by programmatically examining whether a protein had exactly two cysteines and therefore no possibility of misfolding (category 1), more than two cysteines where the disulfide bond was formed between consecutive cysteines (low misfolding probability, category 2), or more than two cysteines where the disulfide bond was form between non-consecutive cysteines (high misfolding probability, category 3).

A master table containing quantity information from the different datasets, numbers of cysteines and disulfide bonds and intracellular protein locations [19–21] was generated by merging the individual data tables listed above (Additional file 1: Table S1).

### Evaluation of proteome coverage

Most of the protein datasets provide information in absolute protein numbers, which need to be converted to concentrations for modelling biochemical reactions. Most of the proteome studies used here do not report cell volume but do report cell growth rates, and the *E. coli* cell volume is known to vary linearly with growth rates [29]. We used two publications that report both cell size and growth rate data [13, 30] to create a conversion factor for estimating cell sizes from reported growth rates. Equation 1 is based on growth rates between 0.1 and 1.9 h^−1^ and was used for estimating quantitative protein coverage. Equation 2 is based only on growth rate values between 0.1 and 1 h^−1^ and was used for converting concentration values to protein count per cell values.

1 $$cell\;size \left[{\upmu}{\text{m}}^{3}\right]=1.44 \cdot growth\;rate \left[{\text{h}}^{-1}\right]+1.90$$

2 $$cell\;size \left[{\upmu}{\text{m}}^{3}\right]=1.83 \cdot growth\;rate \left[{\text{h}}^{-1}\right]+1.74$$

### Estimation of Dsb enzyme abundance

Abundance data for DsbA, C and G were directly extracted from the proteomics datasets via their Uniprot IDs (DsbA, P0AEG4; DsbC, P0AEG6; DsbG, P77202). DsbB and DsbD are membrane-anchored proteins and the membrane association likely leads to depletion of these proteins during sample preparation. Abundance of these proteins was therefore estimated by comparing their synthesis rates inferred from ribosome-profiling based data set by Li and colleges [16]. We assumed that the ratio between synthesis rate and steady-state protein abundance is similar for all Dsb proteins, and there for calculated apparent DsbB/DsbD abundance from their synthesis rates, based on the observed synthesis rate/abundance ratio for DsbA and DsbG.

### Kinetic modeling and parameters estimation

Ordinary differential equation (ODE) models were created using the complex pathway simulation software Copasi [31]. These models were imported into python using the tellurium library [32] and converted to the human-readable antimony model script using pycotools3 [33]. The same package was used to simulate model behaviour over time. The resulting data was processed, analysed and displayed using the Python libraries pandas, matplotlib [34] and seaborn [35]. The iterative loops for identifying minimal kinetic parameter sets were also created using the same python libraries.

### Estimating minimal enzyme activity required for proteome maintenance

Each proteome has a set of kinetic values that need to be achieved in order to satisfy the cells reported doubling time. The kinetic values are gradually reduced until either substrate accumulation exceeds a certain threshold (0.5% per substrate species) or the theoretical proteome doubling time reaches an 5% increase compared to the reported doubling time of the proteome.

## Supplementary Information

**Additional file 1: Data tables**. Details of disulfide bonded proteins in the *E. coli* proteome. Background colours distinguish data from the seven individual data sources used.

**Additional file 2**: **Figure S1**. Analysis of quantitative proteome coverage. Calculations based on cell size estimate based on growth rate (eq. 1, main text). Reported total protein counts are compared to the theoretical total protein count derived from the calculated cell sizes and total protein estimate for *E. coli* by Milo Ren (2013).

## Data Availability

The original source data used for this research was collected from the referenced publications or the uniport database and can be requested from the authors upon reasonable request. The research results and data used in this article have been included in the manuscript and the Additional file.

## References

[1] Bardwell JCA (2007). Disulfide bond formation enzymes. Enzymes.

[2] Berkmen M (2012). Production of disulfide-bonded proteins in *Escherichia coli*. Protein Expr Purif.

[3] Derman AI, Prinz WA, Belin D, Beckwith J (1993). Mutations that allow disulfide bond formation in the cytoplasm of *Escherichia coli*. Science.

[4] Prinz WA, Åslund F, Holmgren A, Beckwith J (1997). The role of the thioredoxin and glutaredoxin pathways in reducing protein disulfide bonds in the *Escherichia coli* cytoplasm. J Biol Chem.

[5] Bessette PH, Åslund F, Beckwith J, Georgiou G (1999). Efficient folding of proteins with multiple disulfide bonds in the *Escherichia coli* cytoplasm. Proc Natl Acad Sci USA.

[6] Gaciarz A, Khatri NK, Velez-Suberbie ML, Saaranen MJ, Uchida Y, Keshavarz-Moore E (2017). Efficient soluble expression of disulfide bonded proteins in the cytoplasm of *Escherichia coli* in fed-batch fermentations on chemically defined minimal media. Microb Cell Fact.

[7] Taniguchi Y, Choi PJ, Li GW, Chen H, Babu M, Hearn J (2010). Quantifying *E. coli* proteome and transcriptome with single-molecule sensitivity in single cells. Science.

[8] Valgepea K, Adamberg K, Nahku R, Lahtvee PJ, Arike L, Vilu R (2010). Systems biology approach reveals that overflow metabolism of acetate in *Escherichia coli* is triggered by carbon catabolite repression of acetyl-CoA synthetase. BMC Syst Biol.

[9] Arike L, Valgepea K, Peil L, Nahku R, Adamberg K, Vilu R (2012). Comparison and applications of label-free absolute proteome quantification methods on *Escherichia coli*. J Proteomics.

[10] Wiśniewski JR, Rakus D (2014). Multi-enzyme digestion FASP and the 'total protein approach'-based absolute quantification of the *Escherichia coli* proteome. J Proteomics.

[11] Peebo K, Valgepea K, Maser A, Nahku R, Adamberg K, Vilu R (2015). Proteome reallocation in *Escherichia coli* with increasing specific growth rate. Mol BioSyst.

[12] Soufi B, Krug K, Harst A, Macek B (2015). Characterization of the *E. coli* proteome and its modifications during growth and ethanol stress. Front Microbiol.

[13] Schmidt A, Kochanowski K, Vedelaar S, Ahrné E, Volkmer B, Callipo L (2016). The quantitative and condition-dependent *Escherichia coli* proteome. Nat Biotechnol.

[14] Milo R (2013). What is the total number of protein molecules per cell volume? A call to rethink some published values. BioEssays.

[15] Kongpracha P, Wiriyasermkul P, Isozumi N, Moriyama S, Kanai Y, Nagamori S (2022). Simple but efficacious enrichment of integral membrane proteins and their interactions for in-depth membrane proteomics. Mol Cell Proteom.

[16] Li GW, Burkhardt D, Gross C, Weissman JS (2014). Quantifying absolute protein synthesis rates reveals principles underlying allocation of cellular resources. Cell.

[17] Roos G, Messens J (2011). Protein sulfenic acid formation: from cellular damage to redox regulation. Free Radical Biol Med.

[18] Bateman A, Martin MJ, Orchard S, Magrane M, Agivetova R, Ahmad S (2021). UniProt: the universal protein knowledgebase in 2021. Nucleic Acids Res.

[19] Lu S, Fan SB, Yang B, Li YX, Meng JM, Wu L (2015). Mapping native disulfide bonds at a proteome scale. Nat Methods.

[20] Chen ZL, Meng JM, Cao Y, Yin JL, Fang RQ, Fan SB (2019). A high-speed search engine pLink 2 with systematic evaluation for proteome-scale identification of cross-linked peptides. Nat Commun.

[21] Loos MS, Ramakrishnan R, Vranken W, Tsirigotaki A, Tsare E-P, Zorzini V (2019). Structural basis of the subcellular topology landscape of *Escherichia coli*. Front Microbiol.

[22] Nath K, Koch AL (1970). Protein degradation in *Escherichia coli*: i measurement of rapidly and slowly decaying components. J Biol Chem.

[23] von der Haar T (2008). A quantitative estimation of the global translational activity in logarithmically growing yeast cells. BMC Syst Biol.

[24] Darby NJ, Creighton TE (1995). Catalytic mechanism of DsbA and its comparison with that of protein disulfide isomerase. Biochemistry.

[25] Karyolaimos A, Ampah-Korsah H, Hillenaar T, Mestre Borras A, Dolata KM, Sievers S (2019). Enhancing recombinant protein yields in the *E. coli* periplasm by combining signal peptide and production rate screening. Front Microbiol.

[26] Hatahet F, Nguyen VD, Salo KEH, Ruddock LW (2010). Disruption of reducing pathways is not essential for efficient disulfide bond formation in the cytoplasm of *E. coli*. Microbial Cell Factories.

[27] Harris CR, Millman KJ, van der Walt SJ, Gommers R, Virtanen P, Cournapeau D (2020). Array programming with NumPy. Nature.

[28] McKinney W. Data structures for statistical computing in python. Proceedings of the 9th python in science conference. 2010 56–61.

[29] Schaechter M, Maaloe O, Kjeldgaard NO (1958). Dependency on medium and temperature of cell size and chemical composition during balanced growth of salmonella typhimurium. J Gen Microbiol.

[30] Volkmer B, Heinemann M (2011). Condition-dependent cell volume and concentration of *Escherichia coli* to facilitate data conversion for systems biology modeling. PLoS ONE.

[31] Hoops S, Sahle S, Gauges R, Lee C, Pahle J, Simus N (2006). COPASI–a complex pathway simulator. Bioinformatics.

[32] Choi K, Medley JK, König M, Stocking K, Smith L, Gu S (2018). Tellurium: an extensible python-based modeling environment for systems and synthetic biology. Biosystems.

[33] Welsh CM, Fullard N, Proctor CJ, Martinez-Guimera A, Isfort RJ, Bascom CC (2018). PyCoTools: a python toolbox for COPASI. Bioinformatics.

[34] Hunter JD (2007). Matplotlib: a 2D graphics environment. Comput Sci Eng.

[35] Waskom ML (2021). Seaborn: statistical data visualization. J Open Source Software.

