# Supplementary figures and images for: A quantitative interpretation of oxidative protein folding activity in Escherichia coli
Source: Microb Cell Fact. 2022 Dec 22;21:268. doi: 10.1186/s12934-022-01982-3 (PMC9773447; doi:10.1186/s12934-022-01982-3)

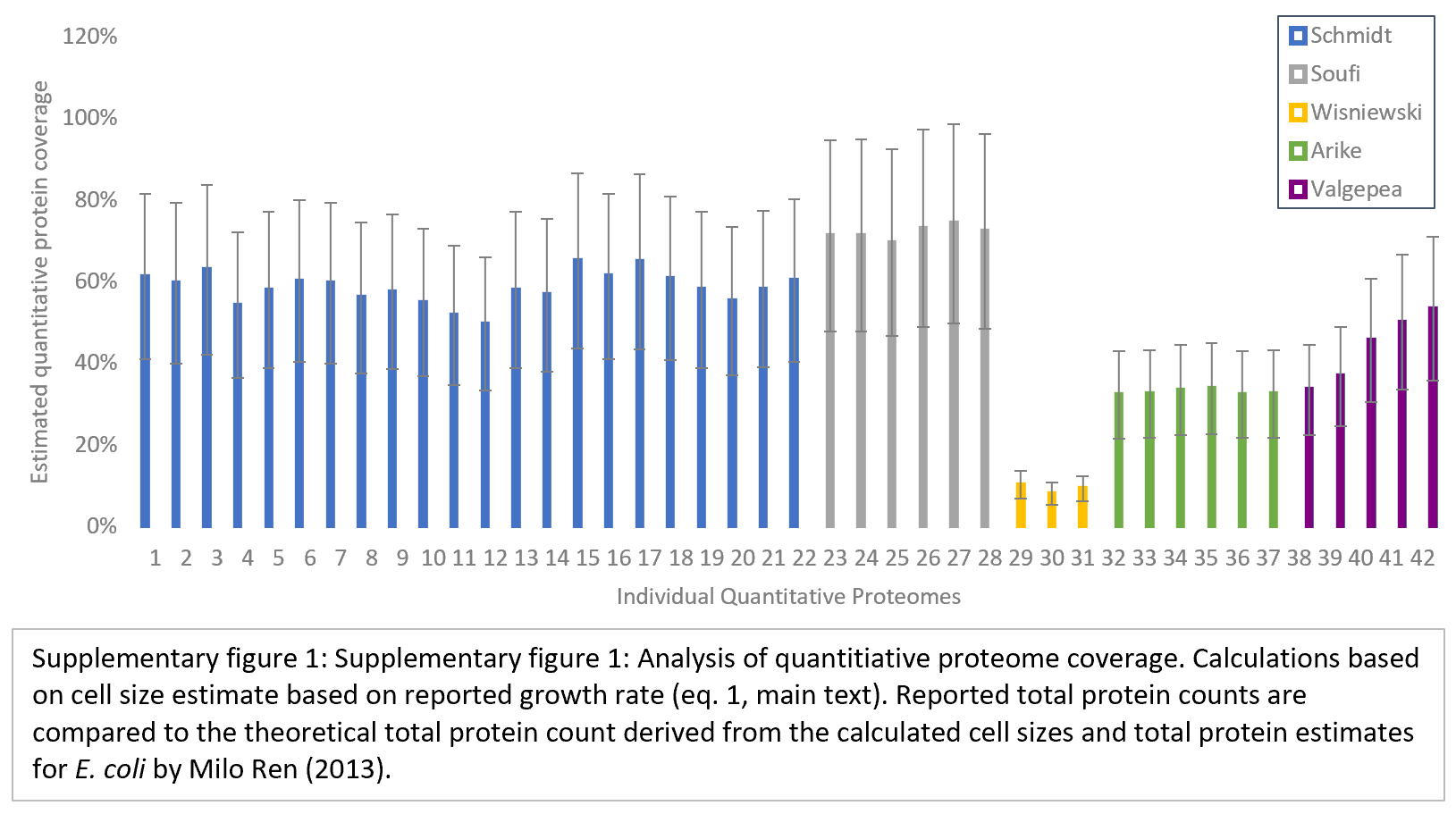

Supplement: Supplementary file 2 — Additional file 2: Figure S1. Analysis of quantitative proteome coverage. Calculations based on cell size estimate based on growth rate (eq. 1, main text). Reported total protein counts are compared to the theoretical total protein count derived from the calculated cell sizes and total protein estimate for E. coli by Milo Ren (2013). [file 12934_2022_1982_MOESM2_ESM.png]
